# Conductive Anesthesia*This article is an abstract from a most valuable paper, read before the New York First District Society, and is printed in full with illustrations, in the *Journal of Allied Societies* for March, 1916.

**Published:** 1916-05

**Authors:** J. E. Nyman


### CONDUCTIVE ANESTHESIA.*

BY J. E. NYMAN, D.D.S.

Any method of local anesthesia or of analgesia must meet the following requirements, if it is to be universally accepted by the profession and their patients:

It must be safe.

It must be certain.

It must be uniform.

Its administration must not be distressing.

Anesthesia must be promptly established.

It must be readily maintained for an hour if necessary.

The patient must be conscious and unalarmed that we may have control of the head and the jaw.

There must be no dangerous or distressing after-effects either locally or systematically.

At present there are two methods which meet these requirements, and are most generally employed by practitioners who systematically endeavor to eliminate pain from dental operations—viz.: analgesia by the administration of nitrous oxid and oxygen and Conductive (also termed by many ''nerve blocking'') or local anesthesia by novocain and suprarenin, or other of the local anesthetics.

### NITROUS OXID AND OXYGEN ANALGESIA AND ANESTHESIA.

Before the writer became conversant with and clinically proficient in the technique of Conductive Anesthesia, his recourse was the nitrous oxid and oxygen analgesia and anesthesia; but some years of experience found him not wholly satisfied with the results obtained, for the following reasons:

The anesthetic must be continuously administered during the operation.

* This article is an abstract from a most valuable paper, read before the New York First District Society, and is printed in full with illustrations, in the *Journal of Allied Societies* for March, 1916.

The patient must inhale in a specific manner continuously, *i. e.*, with a sniffing inspiration through the nose. Many slightly neurasthenic patients could not control and continue this definite specific inspiration, and so the desired analgesia could not be obtained and maintained.

A number experienced thumping pulsations intra-cranially with such apprehensive distress as to render them slightly hysterical.

Some became alarmed by the sense of constriction in the larynx and vocal chords, and this alarm was increased when on attempting to inform the operator of this particular distress the patient found that speech was difficult and impaired and their tones utterly unnatural.

With some, the analgesic state could not be established until the anesthetic state was nearly reached, and they frequently passed into anesthesia with complete loss of control of head and jaw, while the operator was endeavoring to maintain analgesia.

Many developed an analgesic tremor which made careful and finished operating extremely difficult and occasionally impossible. The inhaler was always more or less in the way when operations were attempted on the upper incisors.

The patient's head had to be kept in a fixed position and could not be readily moved without displacing the inhaler and interfering with the administration of the anesthetic.

Some patients developed such an excitement in the analgesic state as to render operating entirely impossible, especially if startled by an unexpected noise or abrupt motion on the part of operator or anyone in the room.

A number experienced such prolonged unpleasant after-effects, accompanied with a sensation of nausea, that they declined to permit this method to be employed again.

I have submitted to this method a number of times and always experienced the very unpleasant intra-cranial effects, and in my case the analgesic state was so close to the anesthetic state, that from time to time I was for a few moments entirely anesthetized.

I have frequently watched clinical demonstrations by operators that were admittedly experienced and skillful, and have noticed all the objectionable features ensue that have been mentioned. Several times I have heard competent observers remark, "Yes, he (the clinician) was able to make some holes in the teeth without pain to the patient, but it wasn't cavity preparation, and he couldn't do it under the circumstances."

Riethmüller expresses a regret in his articles, entitled "Anoci-Association in Dentistry," as follows: "While the voices deprecating the use of nitrous oxid and oxygen anesthesia and analgesia are growing in number and volume in the dental camp—the medical profession having gradually overcome within recent years their prejudice toward anything that savors of dentistry, are becoming more and more enthusiastic about the safety, comfort and efficiency of nitrous oxid and oxygen anesthesia."

This apparent attitude that he regrets is due, I think, not to a lack of appreciation on the part of the dental profession of the laudable properties of $N_2O$ and O, but to an appreciation of the difficulties before mentioned. Is due further to the fact that prolonged dental, oral and pharyngel operations and surgical operations elsewhere on and in the body are themselves so dissimilar. In operations in fields other than the nose, mouth and throat, the mask inhaler covering nose and mouth can be employed, and the desired and requisite percentages of nitrous oxid, oxygen and air can be definitely controlled and maintained; whereas, except for brief operations in the throat and oral cavity, such as simple extractions, abscess lancing and uvula amputations, prolonged anesthesia by nasal inhalers with the mouth wide open for varying ingress of air is not always satisfactory. In prolonged analgesia, which requires a nicety of anesthetic administration, with an exact coöperation of the patient as to special inhalation through the nose; the uncontrolled variation of air content in the anesthetic by reason of involuntary or careless mouth breathing frequently results in an unsatisfactory and disturbing semi-analgesia; while often in operations in certain regions of the mouth the nasal inhaler obstructs the fingers and instruments of the operator.

These are the reasons that explain the difference in attitude of the dental and the surgical professions towards nitrous oxid and oxygen. I occasionally use nitrous oxid and oxygen anesthesia for simple extractions and opening abscesses with great tumefaction and extensive area of infection; and administer it for uvula amputation and paracentesis of the tympanum; but in the place of analgesia for dental operations, I have come to prefer Conductive or local anesthesia.

The objection to the length of time involved in Conductive Anesthesia is due partly to the fact that few dentists, even after clinical instruction, make complete and definite preparation to incorporate this method among their routine methods of practice. If equipped with proper instruments and accessories and with partially prepared solutions, Conductive Anesthesia proves to be an economizer instead of a waster of time.

### CONDUCTIVE ANESTHESIA.

After an experience of over a year and one-half with over 1,500 anesthesias by Conductive and local anesthesia with Novocain-suprarenin involving the greatest variety of cases, both dental and surgical, the writer has come to regard this method as the method ''par excellence'' for the elimination of pain in dental operations for the following reasons:

Under the technique as formulated by Fischer:

It is safe.

It is invariably effective.

It does not require continuous administration during the operation.

It does not require the active coöperation of the patient. tient.

The injections are quickly made and once they have been properly made there is nothing that the patient can do that will prevent the establishment of complete regional anesthesia.

There is no deleterious after-effect either on the pulps of the teeth or the tissues, hard and soft, of the mouth and jaws.

There is no anesthetic appliance obstructing the hands or arms of the operator.

The patient is conscious and has control of the movement of head and jaw and can readily shift the position of either as desired for convenience of operating.

Patients readily and willingly submit to it again and again and commend the method to others.

### THE ANESTHETIC SOLUTION.

The anesthetic solution must be isotonic—that is its salts content must correspond closely to the salts content of the blood and the tissue fluids. This varies from 0.6 to 0.9 per cent. with 0.75 per cent. as what may be considered an average. The physiological salts content varies between the figures given in different individuals and at times in the same individual.

A solution with more than 0.9 per cent. will be hypertonic and a solution of less than 0.6 per cent. will be hypotonic. Solutions which are hyper- or hypo-tonic are apt to give rise to cell disturbances which may cause considerable pain at the time of injection and result in tissue lesions afterward. If solutions of 1 to 2 per cent. of the anesthetic agent are to be used, an isotonic medium obtained by dissolving 1 B. Ringer tablet in 10-cc of pure distilled water may be employed; this gives a 0.56 per cent. salts solution which, with the addition of the anesthetic agent, is raised to the equivalent of 0.72 per cent. to 0.86 per cent. salt solution, according to whether a 1 or 2 per cent. solution is used.

The Ringer B tablet contains Sod. Chlor. .05 grm., Calcium Chlorid .004 gm. and Pot. Chlor. .002 gm. Owing to the calcium chlorid the tablets are exceedingly hygroscopic and if the tube containing them is not carefully sealed and in a dry place, the tablet will deliquesce.

Sterile distilled water, which shows no signs of even minute flocculent particles and which has no trace of alkali must be used. Even a slight trace of alkali will cause a speedy deterioration of the suprarenin and to a slighter degree of the novocain in the anesthetic solution, when boiled. If deterioration of either of these agents has occurred before the injection of the solution, its injection will result in subsequent inflammation of tissues—sometimes of great severity.

The distilled water should be obtained fresh, at least every third day, and kept in one of the Jena Flasks, to avoid an alkaline contamination. The Ringers Sol. is also made every third day and this solution is sterilized by placing the Jena Flask in the boiling water of an electric sterilizer for twenty minutes. The flask should be marked at the 50-cc level, so that the amount of solution evaporated in boiling may be replaced with sterilized distilled water, otherwise the solution would be hypertonic.

With the Ringer B solution as a media the anesthetic agents Novocain and Superarein are added.

Novocain has been chosen by the most noted investigators and clinicians, both in surgery and dental surgery, as the best local anesthetic agent; it is almost equal to cocain in its anesthetizing effect when injected, while its toxicity is but one-seventh of that drug. It produces no tissue lesions and does not deteriorate upon boiling and is not irritant to submucous or subcutaneous tissue.

Cocain was the first drug which came into use for local anesthetic purposes. Owing to many severe cases of collapse, and the number of deaths that occurred following its use, the pharmacological chemists sought to discover a substitute for it. Some two dozen drugs, most of them synthetic, have at various times been offered as substitutes. It is not necessary here to enter into details of description concerning them.

Many of them deteriorate upon effort to sterilize their solution by heat, and many of them produce marked pain immediately after their injection and before the anesthetic effect is obtained; while a number of them give rise to more or less serious tissue inflammations after the anesthetic effect is past. Braun, Seidel and Réclus have found that cocain, B. eucain and novocain are the only local anesthetics, injections of which do not produce pain, and do not give rise to secondary tissue inflammations. B. eucain does not exceed novocain in anesthetic properties, while its toxicity is from two and a half to three times that of novocain.

Kocher of Switzerland, Braun, Fischer, Narath, Seidel, Wilms, Axhausen, Hesse, of Germany; Arnold, Struthers, Le Brocq of England; Réclus, Piqquand of France; and the Mayos, Crile, McArthur, Cushing, Harris, Schley and Matas of America, are among the notable men who employ local anesthesia largely in operations, and after clinical experimentation with the various local anesthetics, have chosen novocain as the anesthetic for their use.

The use of quinine and urea hydrochlorid in connection with epinephrin, has been suggested; competent investigators, however, have observed that solutions of these agents cause painful injections and give rise to quite marked tissue inflammation later, while the anesthetic effect can not compare with the three agents mentioned.

Suprarenin, a synthetic preparation, similiar to adrenalin in its action but of greater stability, is added to the solution because it intensifies and prolongs the regional or local anesthesia; its contractile effect upon the blood vessels in the neighborhood of the point of injection, retards the absorption of the anesthetic solution into the general circulation.

Suprarenin deteriorates quickly after exposure to moisture, air and heat. If there is any alkalinity in the Ringer solution, the solution turns pink and later, as further deterioration occurs, brown. Such solutions should never be injected. If they are, a local toxemia is apt to result, resembling the lesions which occurred in so many instances in the past when hydrate of chloral injections were used.

Suprarenin solutions should not be kept in stock. The drug keeps well in dry form only. Ringer's B. solution may be kept in stock, but the anesthetic solution should be prepared at the immediate time of injection. Any residue of anesthetic solution not injected should be expelled from the syringe at once.

Tablets containing novocain and suprarenin according to formulæ of Prof. Fischer have been prepared by the Farberwerke Hoechst Company. Tablet E. containing novocain .02 gm. and suprarenin, .00005 gm. Tablet G. containing novocain .015 grm., suprarenin, .00005 gm. These tablets in the ratio of 1 tablet to each c.c. of Ringer's Sol. give a 2 and 1½ per cent. anesthetic solution respectively.

Suprarenin is one of the most powerful drugs known and one of the most active in both local and systemic effect. In some patients its action has been exhibited after the injection of less than 1 c.c. of a 1 :100000 solution.

Frequently in plethoric, nervous or anemic patients, also in the very young or very old, after injections in usual quantities of solutions made from either E. of G. tablets, a reaction follows within from ten to thirty seconds, of variable duration, but always transitory. This reaction, if marked, is often alarming and distressing to the patient. Pulse-rate and blood pressure are increased, a tremor develops, usually slight but sometimes increasing to a palsy so that the patient can not hold a glass. There may be a sensation of nausea and a cold sudoresis with marked pallor; in hysterical individuals there may in addition supervene, syncope and hysterical spasms.

As I have said, these reactions are transitory, and while alarming, are not dangerous, and I believe with Thoma that they are due to the effect of the suprarenin and not that of the novocain.

The writer is of the firm conviction that the reactions which are not hysterical and psychological, are due almost entirely to the action of the suprarenin and his personal opinion is identical with the conclusions expressed in the resolutions adopted by the meeting of the Local Anesthetists held at Münster in 1912, part of which are as follows:

"Sec. 10—The optimum concentration of novocain for dental purpose is 2 per cent.

"Sec. 11—On the other hand the concentration of suprarenin calls for individualization in many cases in dental practice; this is necessary because the toxicity of the suprarenin depends largely on the concentration of the dose, especially in aged individuals and arterio-sclerotics and in case of accidental injection into blood vessel, etc.

"Sec. 12—In normal cases the best results are obtained by the addition of .00002 gm. of suprarenin to each c.c. of 2 per cent. novocain solution.

"Sec. 13—In cardiacs and aged this dosage should be reduced to .00001 gm. if pronounced anemia in the field of operation is desired, increased to .00005 gm."

It is quite true that even if 10 c.c. of anesthetic solution containing .00005 gm. to each c.c. were injected the patient's life would not be endangered. It is also true that in hospitals in cases of collapse, from .005 gm. to .02 gm. are administered in 24 hours. Therefore the maximum dosage that would be administered for dental or oral surgical operations may be considered safe if the solutions are sterile and chemically unchanged. But the hospital cases are cases in collapse, while we are considering cases that are normal or slightly stimulated from pain or apprehension and in advocating a reduction of the suprarenin content it is not from a consideration of avoiding danger, but of avoiding distress, mental and physical, even though they are but transitory.

Fischer in his book recommends the use of the 1 per cent. and 1.5 per cent. solutions in ordinary dental operations; these if made by dissolving 1 E. tablet to each 2 c.c. of solution or 2 E. tablets to 3 c.c. will result in 50 per cent. less suprarenin in the 1 per cent. solution and 25 per cent. less in the 1½ per cent. The writer finds that these two percentages are effective for all classes of dental operations. For the removal of roots that are ankylosed in sclerosed bone, removal of impacted 3d molars, removal of cysts and osseous tumours, root amputations, etc., a 2 per cent. solution is used; made by dissolving one E. Tablet to each c.c. of solution. This gives, besides profound anesthesia, an anemia to the field of operations which makes the operation more rapid and certain.

Anesthetic solutions should not be kept on hand. Stale solutions, even though sterile, have been known to cause tissue lesions and since anesthetic solutions are so quickly prepared, if sterile Ringer's solution is on hand, there is no adequate reason for using stock anesthetic solutions.

In cases where there is reason to fear long continued post operative pain, as in the removal of badly impacted third molars, a prolonged anesthesia may be established by adding 15 per cent. alcohol to the second syringeful of anesthesic solution injected at the mandibular foramen.

I have noted anesthesia which persisted for two or three weeks following these injections, which did not, however, interfere with the healing process, nor did they result in any tissue lesions at the place of injection.

### PRE-OPERATIVE PREPARATION OF THE PATIENT.

To lessen the likelihood of reaction from the suprarenin, the writer believes it is best to avoid, if possible, instituting Conductive Anesthesia within two hours after a meal. If one from the necessity of practice, must for example, appoint the hour of 10 a. m., or 2 p. m., for Conductive Anesthesia, the patient should be advised to take a light breakfast before eight or a light luncheon before twelve.

In extremely excitable or apprehensive patients one or two tablets of bromural, or one or two tablets of codeine phosp. gr. 1/4 may be given one-half hour before injecting; especially to those of this type who are to have Conductive Anesthesia for the first time; usually it is not necessary in succeeding instances. The phosphate of codeine is used because the patient can be told if insistently inquisitive that you have given them an alkaloidal phosphate.

I have not found it necessary as yet to administer morphin or scopolamin and morphin.

It is also advisable to see that nothing occurs in the pre-injection period that is startling or repugnant to the patient. Everything about operator and assistant, about the equipment and instruments should suggest cleanliness. Allow the patient to watch the boiling of the solution and the sterilizing of the needle in the flame, at the same time informing him that this prevents any danger of infection.

Let there be a calm and confident manner about the operator's movements and speech, as if to suggest that the proceeding is a matter of fact, everyday method of your practice, over which there is no reason to become excited, and which does not give you any concern beyond care as to cleanliness.

Then with deft and precise manipulation, avoiding false motions, the injections may be accomplished without alarming the patient. The writer advises that a thin rubber finger cot be worn on the index finger of the left hand; this not only is a guard against infection of the needle, if it come in contact with the palpating finger; but also prevents the staining of the finger with the iodine solution, the stale odor of which is disagreeable.

In palpating for the anatomical landmarks, especially for Conductive injections in the mandible, do so in a gentle, deliberate manner, being especially careful not to touch the base of the tongue or the anterior pillar of the pharynx, as in some sensitive patients this will immediately cause a sensation of nausea. This may be avoided by always passing the index finger between the teeth and the cheek and then over the last molar to the retro-molar fossa, which is located slightly buccally to the last molar tooth.

Finally take precaution that the operation shall not be disturbed, either by the ringing of the phone or a knock at the door.

### PREPARATION OF THE FIELD OF INJECTION.

Since the fluids of the oral cavity always contain bacteria in a varying degree of virulence, measures must be taken to sterilize the field at the point of injection. Iodin solutions are the most effective agents for this purpose. Fischer suggests a solution of equal parts of tr. iodin, and tr. aconite, which not only disinfects, fixes and dries the mucous membrane, but also, through the aconite agent, causes some local surface anesthesia. But as Riethmüller has stated, iodin solution, unless fresh, may deteriorate in hydriodic acid, which is quite caustic and may result in a disagreeable burn. Furthermore, even fresh tr. iodin may have a caustic effect upon mucosa that have an idiosyncrasy against it.

A 5 to 10 per cent. solution of iothion (colorless iodin) in olive oil, to which has been added menthol 0.7 gm., chloroform 3 c.c. to each 30 c.c. will sterilize the field without danger of caustic effect, even in those cases of idiosyncrasy; the menthol acting as an efficient refrigerant surface anesthetic. Another refrigerant antiseptic that I may suggest, is composed of 2 per cent. solution of iodin crystals in equal parts 70 per cent. alcohol and tinc. benzoin to which is also added 2 per cent. of menthol. This solution should be freshly made every seven days to avoid any development of hydriodic acid.

Prior to applying the antiseptic a cotton roll should be placed between the cheek and the upper molars to block the flow of saliva over the point of injection, the field is wiped off before applying the refrigerant antiseptic, and after this has been applied (avoiding a surplus of antiseptics), the menthol is evaporated by a gentle stream of air from the chip-blower, the point of which has been held in the flame while filling it with air. This evaporation produces a refrigerant anesthesia that minimizes the sensation of the initial penetration of the needle. In regions which are accessible for spraying with chlorid of ethyl, as in the mucous membrane over the anterior teeth and the post-zygomatic region; a refrigerant anesthesia can be produced by this ether spray that will enable one to make a painless insertion of the needle. This measure can not be applied, however, to the ramus, as the vapour produces spasmodic coughing, when the spray is directed into the region of the pharnyx.

### THE VARIOUS METHODS OF INJECTIONS.

As to the methods of making the injections, there is to be considered the question of Conductive Anesthesia or infiltration anesthesia.

My choice is for Conductive Anesthesia, in both maxilla and mandible for the obtunding of sensitive dentin or for pulp removal. Injections in the pterygo-mandibular space along the ramus, in the post-zymogatic and the infraorbital regions are accomplished with less pain at the time of injection, the anesthesia is more certain and definite and there is less soreness during the next 12 to 72 hours, than in local submucous injections, as in these regions loose connective tissue alone is encountered, and but little force in injecting is necessary.

Submucous injections, because of the increased pressure needed to force the solutions into the periosteal regions by reason of the resistance of the dense fibrous connective tissue of the gums, are more painful at the time of injection and there may be some soreness at the regions injected during the succeeding days, due simply to mechanical irritation and quite without septic or chemic cause. These submucous injections alone are not so definite and certain in producing anesthesia, as osseous infiltration will depend on the porosity of the cortical layer of the bone tissue.

Intraosseous injections, with but slight submucous injections in certain fields, produce definite anesthesia and with little pain and there is little probability of subsequent regional soreness.

INTEROSSEOUS INJECTIONS.

The mucosa is anesthetized over a small field by slight submucous injections, then with a sterilized ½ round bur or drill in the handpiece, the gum and cortex of the bone is pierced and a minute hole drilled into the cancellous portion of the bone. Into this opening is placed a short square pointed needle and about .25 c.c. of 1 per cent. to 2 per cent. anesthetic solution is injected. This is quickly diffused through the spongiosa of the bone to the pulp and pericementum, resulting in quite complete anesthesia of one or two teeth in a very short time.

This method was suggested and advocated by Otto and Parrott in about 1896 and later by Masselink. They suggested drilling at a point directly opposite the apex of the tooth to be obtunded, but, as Fischer has pointed out, you can not be certain of this location unless a clear and accurate radiograph is at hand; furthermore, in many instances, at the reflection of the mucous membrane, there is but a thin layer of bone over the root and the operator may inadvertently drill through not only the cortex, but the pericementum and cementum and then make a futile effort to inject into the dentin of the tooth. The writer saw one clinical demonstration of this method that failed, from what he believes to have been this cause, although the demonstrator ascribed it to "thickening of the bone," but, from the evidence of pain exhibited by the patient during the last half of the drilling, I feel quite sure that the pericementum, cementum and dentin of the tooth were invaded.

After some experience with the method, I would make the following suggestions: That it be confined to the region of the incisors and first bicuspids, as this field can most surely be kept from the flooding by mucous and saliva after sterilizing and during the drilling and the substitution of the blunt needle for the drill. That the point of insertion of the drill be the interproximate space on both sides of the tooth to be anesthetized and about midway between the gingiva and the reflection of the mucous membrane; then there is no danger of laceration of the loose mucosa by its wrapping about the revolving drill, or of perforating the pericementum, cementum and dentin; damaging structures, the last two of which have very little reparative power; and causing pain which by flinching of the patient may result in the breaking of the drill, leaving it imbedded in a region where its removal is extremely difficult and likely to result in still more serious damage to the tooth. In drilling at the points suggested by the writer, there is little danger of such an accident, as no pain is caused. The cortex repairs rapidly if no infection is introduced during this operation.

This method is more particularly advantageous in the lower jaw, as the cortex of the mandible is thicker and has fewer foramina than has the maxilla.

### PERIDENTAL INJECTIONS.

Prinz suggests the direct injection of the peridental membrane by inserting the needle between the tooth and the free margin of the gum, directly into the peridental membrane. If the tooth is in proximal contact with adjacent teeth, it is to be wedged in a distal direction while the mesial injection is made, then wedged in a mesial direction while the distal injection is made. He states this method produces speedy anesthesia of the tooth, and is especially serviceable for removal of pulps or temporary desensitizing for cavity preparation in cases where pressure infiltration through the cavity wall or floor is contra-indicated.

He advises against the procedure if there is found acute inflammatory conditions of the peridental membrane. The objections to this method are—danger of infection in cases where obscure pyorrheal conditions exist; the strong pressure necessary to inject this tense and highly sensitive membrane produces pain which continues until anesthesia is developed.

The circulation of the peridental membrane is seriously disturbed and because of the anatomical environment it tolerates badly, recovers with difficulty and sometimes fails to recover from such disturbance; moreover, the tooth is usually very sore for days after the anesthesia has passed.

As Fischer sums it up "The wedging of the tooth, the insertion of the needle and the force necessary in injecting are almost as painful as the operation would be without the local anesthesia."

In justice to Dr. Prinz, and in view of his excellent chapter on "Local Anesthesia" in Johnson's "Text Book of Operative Dentistry," it must be pointed out that he suggests but does not emphatically advocate this method.

### REACTIONS.

The immediate reaction that occurs occasionally is due, I believe, to the action of the suprarenin; to forestall apprehension, which always aggravates the reaction, the patient is informed at once the injection is made, that there may be a little increase in the heart action, and they may feel "shaky" generally, but that this will pass in a few moments. If this reaction becomes marked, the patient is given validol camphoratum .5 c.c. (eight drops in 15 c.c.) (one tablespoonful) of water, or 2 c.c., thirty drops aromatic spirits of ammonia in 30 c.c. (one ounce) of water, or .75 c.c. (twelve drops) of spirits of camphor in 15 c.c. of water.

In severe reactions with syncope 1 to 2 c.c. camphorated oil or nitro-glycerine, 1-50th grain may be administered hyperdermically, or amyl nitrite inhalations may be used.

I have seen but one severe reaction; this case (a young man of slightly hysterical temperament) occurred in one of Fischer's clinics. Marked pallor, stertorous breathing, rapid pulse reaction quickly developed into tonic spasm. He twisted partly out of the chair onto the floor; while we were endeavoring to replace him in the chair to inject camphorated oil the spasm ceased, he regained consciousness and quickly recovered his normal condition. He was greatly surprised to learn that anything unusual had occurred. He had been conscious of a slight chill and nothing more. After being operated on, without the slightest sensation of pain, he asked if he might remain a while, and so enthusiastic was he that he remained about an hour, reassuring the other patients that it was painless and not to be feared.

There have been authentic reports of instances of brief narcosis with quite realistic dreams, and for the protection of the operator's reputation, it is advised always to have a third person present.

Secondary reactions occurring from the following day up to the third day thereafter are due to either deterioration of the novocain-suprarenin in a stale solution or to infection from careless technique, and either of these reactions may be so serious as to be tragic; therefore the operator must acquire, by earnest self-training, what is known among surgeons as "the aseptic habit."

### TIME SCHEDULE OF ANESTHESIA.

Fischer in his clinical classes stated that ten minutes should be allowed to elapse after injections in the maxilla, and twenty minutes after injections in mandible. This has made many busy operators, including some who have had clinical instruction, feel that they could not make it a routine method of their practice, reserving it for an occasional use in exceptional cases. But it must be remembered that Fischer was demonstrating a comparatively novel method of anesthesia, and that to convince both practitioner and patient that it fulfilled all that was claimed for it, it was essential to be certain that anesthesia was profound before operating; for if the slightest sensation of pain was evidenced, the practitioner would be dubious concerning the efficiency of the method and rather diffident about attempting it.

I have seen many demonstrations of pain elimination by other methods, but I have never seen anything so completely convincing as were the demonstrations of Conductive Anesthesia by Guido Fischer, and this is also the feeling of the score of men who attended the classes held by him in my office.

In surgical operations, in immediate pulp removal, in drilling into an acutely abscessed tooth, it is well to wait until anesthesia is profound; but partial anesthesia is soon established and reduces sensation to the point where it is comparatively tolerable, so that in cavity preparation the preliminary operations may be begun within a few minutes after the injections are completed, and, by the time the especially sensitive regions must be cut into, anesthesia will be complete.

If one is properly equipped, with syringes with needles attached suspended in the alcohol solution in the specimen jar, with the refrigerant antiseptic solution, the Ringer's solution, clear distilled water, and the validol camphor mixture in stock, and with the graduated porcelain mixing cups at hand, the following schedule of time may easily be maintained:

Preparation of anesthetic solutions and filling of
syringe . . . . . . . . . . . . . . . . . . . . . . . . . . . . 1 minute.
Sterilizing of field and injecting for Conductive
Anesthesia . . . . . . . . . . . . . . . . . . . . . . . . . . 2 minutes.
Partial anesthesia obtained, in maxilla . . . . . . . . . 3 minutes.
in mandible . . . . . . . 5 minutes.

I have in a large number of cases found complete anesthesia in the maxilla in seven minutes after injecting at the tuberosity, and in the mandible in twelve minutes after injecting at the ramus. My experience, in this regard, corresponds with that related by Fischer. In interosseus injections, I have found complete anesthesia in three minutes after injecting.

The technique has been but summarized, because the purpose of this paper is not so much clinically to instruct you in Conductive Anesthesia as it is to endeavor to convince you that this method is so valuable, so practical, that every competent progressive practitioner should obtain clinical instruction and acquire technical proficiency in it. Its clinical merits alone have made me not only its practitioner, but also its advocate.

Complete explanation of the entire method is to be found in the splendid text books on "Local Anesthesia in Dentistry," by Fischer-Riethmüller, and "Oral Anesthesia," by Thoma and by Lederer; books which should find a place in the library of every dentist who aspires to keep pace with front ranks of his profession.

But, as Riethmüller has suggested, one would not expect a successful laparotomy to be performed, or a successful gold filling to be inserted, by one whose only instruction was that obtained by study of the directions in an ideal text book; neither should one expect to achieve success in Conductive Anesthesia by reading a book on it; witnessing, with a crowd of other observers, a practical demonstration, and then making a few diffident and disappointing attempts to perform it.

Small clinical classes, not to exceed ten men, should be formed, and clinical instruction by an expert in the method arranged for.

Text books are exceedingly valuable for obtaining acquaintance with the method and for refreshing one's memory, but only by clinical instruction and clinical practice will the details of the technique become so familiar as to assure uniformly successful results.

Though Conductive Anesthesia permits rapid operating, this must not be carried to the haste of careless operating. A vital tooth temporarily anesthetized, should not be treated without consideration regarding thermal shock as if it were a devitalized tooth. While the patient feels no pain, the tooth pulp is still susceptible to the effects of irritation and injury of thermal shock, and though, for the time being, the line of information has been blocked, it will register its complaint plainly enough when communication with the brain has been re-established, and perchance, after a period of painful disturbance, succumb altogether.

It is a method that only the careful and competent should practice; at the hand of those who are without either of these attributes it may prove dangerous, injurious and ineffective, reflecting discredit upon both operator and method.

Severe pain for the patient exhausts not only him, but through psychic influence the operator as well. Few men are there in the profession who are so callous that they are immune to the fatigue induced by a patient's suffering.

The mission of the dentist is the prevention and cure of oral and dental disease, the relief of pain and the repair of damage. One of his missions being the relief of pain, I feel that he is no longer justified in causing it, since there are now methods proven safe and certain whereby pain can be entirely eliminated from dental operations. It is not claimed that analgesia or anesthesia should be used in every case that presents itself; there are many brief operations in which local obtundents, sharp instruments, and precise and deft manipulation, reduce the momentary sensation of pain to the point where it is easily tolerable. But there are many, many cases, as you well know, where these measures are of little avail to mitigate severe, intolerable, prolonged periods of pain that exhaust both patient and operator.

The public is fast becoming acquainted with the fact that there are now reputable methods of painless dentistry, and dentists unequipped by knowledge and technical skill to apply these methods can not much longer evade the inquiries of patients, by intimating that they are not safe or efficient.

Among the supremely valuable methods that have been given to our profession during its history, there is none more valuable or beneficient than that of "Conductive Anesthesia."

I can testify that it is a boon to the operator, reducing fatigue, facilitating accurate operating, expediting the time of operation, while to the patient it is a blessed boon that banishes pain from the frequent operations they must undergo and it robs the dental office of its old-time widespread dread.

